# The Effect of Mask Style and Fabric Selection on the Comfort Properties of Fabric Masks

**DOI:** 10.3390/ma15072559

**Published:** 2022-03-31

**Authors:** Adine Gericke, Jiří Militký, Mohanapriya Venkataraman, Hester Steyn, Jana Vermaas

**Affiliations:** 1 Department of Chemistry and Polymer Science, University of Stellenbosch, Stellenbosch 7600, South Africa; agericke@sun.ac.za; 2 Department of Material Engineering, Faculty of Textile Engineering, Technical University of Liberec, 46117 Liberec, Czech Republic; jiri.militky@tul.cz; 3 Department of Sustainable Food Systems and Development, Faculty of Natural and Agricultural Sciences, University of the Free State, Bloemfontein 9300, South Africa; hesterjhsteyn@gmail.com (H.S.); neljf@ufs.ac.za (J.V.)

**Keywords:** fabric masks, COVID-19, mask style, micro-climate, temperature, humidity

## Abstract

The purpose of fabric masks in the prevention of the spread of COVID-19 often requires that the masks be worn for extended periods without removal. The management of the conditions in the micro-climate inside the masks is important to keep the wearer comfortable and enhance user compliance. In this study, the effect of mask design and fabric type on the micro-climate was investigated using thermocron iButtons to record the temperature and humidity inside the masks. It was found that the mask style, and its effect on the amount of air incorporated in the micro-climate, had a significant influence on the factors that determine the temperature and humidity levels. In the shaped masks, the impact of the mask design on the results was stronger than the effect of fabric type. In the folded masks that fit snugly around the face, the effect of fabric type was significant, and both fibre composition and fabric structure contributed to the differences in the performance of the three fabrics tested. In the case of the masks with an inserted filter, a significant amount of trapped still air in the fabric layers and the increased mask stiffness had the strongest effect on the temperature and humidity inside the masks. Significant differences were also found in the temperatures recorded in the different time segments, highlighting the importance of conducting comfort evaluations over a long enough time to prevent false interpretations. The results of this study emphasize the importance of considering all the components of mask design, namely style, fibre type, and fabric structure, in the development of masks to enhance user compliance.

## 1. Introduction

Since the wearing of face masks made of ordinary textile fabrics (generally referred to as “cloth masks”) was recognized as probably the most effective means to mitigate the COVID-19 disease transmission (by preventing outward transmission of contaminated droplets by an infected person) [1,2,3], the wearing of a face mask in public places and the work environment has become part of our lives. It is disconcerting, however, that the image of a face mask moved to a position under the chin or below the nose of the wearer, due to the development of discomfort, is not an uncommon sight.

Fabric face masks are designed to serve as “source control”, providing sufficient filtration efficiency to stop the transmission of micro-droplets (≥5 µm) from an infected person to the environment [4]. To ensure the success of this intervention, the impact of “user compliance” should not be underestimated. The frequent removal (or even touching or adjustment) of a mask, due to discomfort, can compromise its effectiveness significantly. Masks should be designed to fit snugly and to cover the nose and mouth acceptably to minimise the leakage of respiratory excretions. The components should be selected to enhance the thermo-physiological comfort experienced by the wearer to such an extent that the mask can be worn in the correct position for long periods when required.

Comfort in clothing is generally defined as a state where the wearer is unaware of the textiles covering the skin, and the sensation is often expressed as freedom from “pain” or a neutral state [5]. Mild to severe deviations from the neutral state in mask-wearers will lead to frequent touching or displacement of a mask (with the hands) and temporary or permanent removal from the face. Apart from the failure in terms of source control, this could lead to an increase in self-infection or the creation of a route for secondary infection [6].

There is no doubt that, although efficient filtration efficiency and air permeability are important requirements, the design and selection of the material components that determine the level of wear comfort should be prioritised. The thermo-physiological comfort properties in a textile fabric are closely related to its ability to provide thermo-regulation and moisture transportation from the micro-climate (the area between the skin and the first layer of fabric) to the surrounding environment [7,8,9]. This implies that masks that can quickly transfer heat and moisture away from the face are considered to provide higher levels of comfort [10].

Although the physical design and fit of reusable fabric masks have received a great deal of attention, there is a lack of reliable information on the comfort-related factors influencing user compliance. Studies on the wearing of masks by medical personnel show that non-compliance to the recommended practices was mostly due to factors related to the development of a state of discomfort during wear [11,12].

The reported research on mask comfort focuses mainly on the comparison of surgical and N95 masks in the medical environment. Differences in the thermal resistance and breathability, as well as micro-climate conditions (temperatures and humidity), were common among the variety of masks tested. The micro-climate in the thinner, more air- and moisture-permeable surgical masks was less humid and hot than in the N95 masks, leading to significant changes in the subjective perception of comfort. Differences in the measured heart rates, skin temperature, and perceived humidity of participants were reported, causing greater wearing discomfort and lower user compliance [6,13]. It was also found that skin temperature increased irrespective of workload [14]. Cao and Cloud [11] reported that an increase in ambient temperature, heat, and moisture trapped in surgeons’ masks caused a significant rise in the temperature and humidity in the mask-wearers’ facial environment.

Heat loss through the facial area plays an important role in the thermoregulation of the body. Due to the high concentration of thermo-receptors, it accounts for 20 % of the total heat loss drive (cooling action) from the skin. This process is impeded when a mask, covering the nose and mouth, is worn [6]. Changes in the micro-climate in a mask can lead to the development of heat stress [15] or a potential decrease in mental and physical performance [16]. Studies [6,14] reported an increase in the skin temperature around the nose and mouth, and regarded increases of less than 2 °C as significant.

The body temperature is kept constant by a combination of physiological mechanisms, of which the cutaneous thermal receptor impulses from the facial nervous system are more important than those from other regions. This causes an increase in the facial temperature (when induced by the mask) to have a significant effect on the thermal sensations of the whole body [6,14]. It was reported that, when the face of a healthy individual was exposed to heating, the local sweating of a leg was affected three times more than when the heating was applied to the leg itself [6].

As most of the studies mentioned focused on surgical and N95 masks, it is postulated that the effect would be accelerated in fabric masks, where the total thickness and thermal resistance of the masks depend on the individual fabric properties. The fabrics used in fabric face masks include combinations of woven, knitted, and nonwoven fabrics and are generally much thicker than the spun bond or melt blown nonwovens used in medical masks.

Several factors affect the micro-climate inside a mask. Studies have shown that the fabric thickness is a key indicator of thermal resistance [17], with the effect being accelerated when multiple layers are involved. Heat loss through a fabric is not determined solely by the thermal conductivity of the fibres but depends largely on the amount of air incorporated in the fabric structure (hence the thickness) [17,18,19]. This is explained by the fact that air has a thermal conductivity of 0.024 W.K^−1^·m^−1^, which is 10 times less than that of the average textile fibre. This causes air trapped within a fabric structure to have an overbearing effect over specific fibre thermal properties [20]. Jung et al. [21] compared the cup- and pleated-style masks and attributed the differences among the conditions measured inside the masks to the amount of dead air space created between the skin and mask by the mask’s design.

Body heat is conducted from the skin to the layer of air that surrounds it. The heat is then transferred further through the micro-climate and then through the air between textile layers and surrounding individual textile fibres and yarns and, lastly, through the textile fibres. If the design of the mask allows it to make solid contact with the skin, the dry heat loss changes from convective to conductive, which may restrict the insensible heat loss. A higher airflow rate through the mask (more breathability) increased the convective heat transfer from the skin to the environment [8,14].

Moisture management inside the micro-climate involves the transfer of moisture vapour through the air spaces between the fibres and yarns to keep the micro-climate dry enough to allow for the evaporation of sensible and insensible perspiration from the skin surface. In addition, the mask should allow any liquid moisture to be absorbed and spread effectively throughout the fabric, offering a dry hand, and enabling moisture to be evaporated [7,8]. Exhaled air and water vapour from breathing further alter the temperature and humidity levels inside the micro-climate. If the moisture management properties of the fabric are inadequate, the effect can accelerate perspiration. This will result in an uncomfortable sensation of wetness and eventually wetting of the mask, causing unwanted clinging to the face [10].

The importance of maintaining thermo-physiological comfort in the micro-climate between the skin and a mask to enhance user compliance is evident. This study aims to explore the field further by investigating the effect of mask design and fabric structural properties on the temperature and humidity in the micro-climate between the skin and fabric mask.

## 2. Methodology

“Mask design” includes not only the style in which the mask was made but also the textile fabrics that were incorporated in the different layers. This study aims to investigate the effect of all these factors on the thermo-physiological comfort of the wearer. In masks, comfort relates to how well the micro-climate inside the mask is managed in terms of temperature (T_mc_) and humidity (H_mc_). For both T_mc_ and H_mc_, the effect of all aspects of the mask’s design, which include the physical design of the mask (in this study referred to as mask style) as well as the performance properties of all the fabrics the mask is made of, will be considered.

Thermocron iButtons were used to measure and log T_mc_ and H_mc_ in the micro-climate between the skin and the mask. iButtons, commonly used in clinical trials, are small, standalone, calibrated data loggers that can be placed directly on the skin to log temperature and humidity at a user-defined rate. In this study, T_mc_ and H_mc_ were recorded at sixty-second intervals over a minimum duration of thirty minutes under conditions resembling an office, laboratory, or general work environment. Three mask styles and three fabrics/filter combinations were evaluated. The mask styles were selected based on popular designs manufactured and worn regularly by the public. The masks for this study were previously evaluated in a subjective wear trial and were all rated as “acceptable” concerning wear comfort. It should be noted that the study aimed to investigate factors that influence the micro-climate in masks that conformed to the general guidelines for face masks and did not include any masks that were already rated as uncomfortable or did not meet the general requirements for fabric masks.

**Figure 1:**
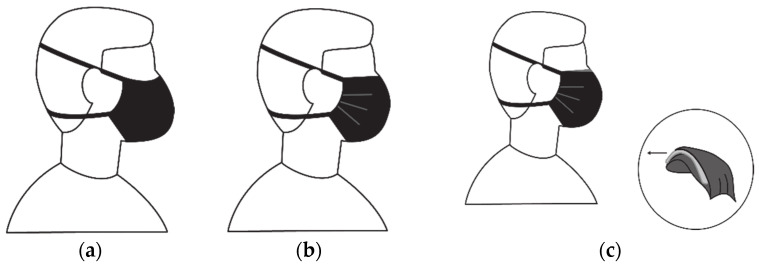
Illustration of mask styles. (**a**) Shaped mask (style S); (**b**) pleated mask (style E); (**c**) mask with filter (style EF).

In this study, the term “mask design” refers to the combination of mask style and fabric type in a specific mask and the combinations used are described in the “mask references” (Table 1). The three mask styles included a two-layer folded style mask (Style E), which is constructed like an envelope with an opening at the top where a filter can be inserted if required. Style EF is a mask with the same design as style E with a filter inserted between the two layers. Style S is a shaped mask consisting of two fabric layers with a thin nonwoven filter permanently stitched in between to keep its shape. The filters used in style EF and style S masks were selected for optimum performance in the specific designs; they differ in thickness and stiffness. The mask styles are illustrated in Figure 1.

**Figure 2:**
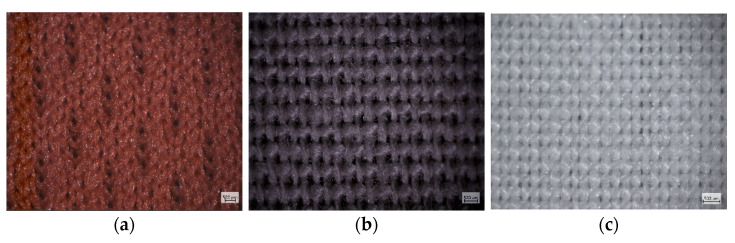
Microscope images of fabrics used as inner and outer layers (scale = 500 µm). (**a**) Weft-knit polyester; (**b**) woven cotton; (**c**) woven polycotton.

The three fabrics (selected as inner and outer layers) and the filter fabrics are described individually in Table 2 and shown in Figure 2. In all the mask styles, the inner and outer layers of a mask were made of the same fabric types. The fabrics were analysed and tested in the laboratory, and selected structural and performance properties that relate to thermo-physiological comfort are summarised in Table 1 and Table 2. Measurements are reported for single, double, and three-layer fabric assemblies. The masks were washed and conditioned for 24 h before testing in the standard environment for conditioning following ISO 139:2005 (20 ± 2 °C, 55 ± 4 % RH) to ensure consistency.

The filtration efficiency (FE) and air permeability (AP) of the selected fabrics were measured by Gericke, et al. [22] using a method that utilizes upstream and downstream particle count measurements to calculate FE (%) and AP (%). Fabric thickness (mm) was measured with a John Bull Imperial Indicator. Fabric weight (g/m^2^) was determined according to SANS 79, using a circular cutter for accuracy. Relative water vapour permeability (WVP) and thermal resistance (R_t_) were determined using the Permetest instrument. The Permetest is a fast response measuring instrument for the non-destructive determinations of the water vapour (%) and thermal resistance (m^2^·K.W^−1^) of fabrics on single or multiple layers of fabric [17,23]. The ability of the fabrics to absorb and wick water (referred to here as horizontal wicking) was measured using a test procedure that was developed based on the method described in FTTS-FA-004. In the revised method, specimens are mounted on a ring (20 cm diameter), with the face side up, and an amount of 0.2 mL of distilled water is dropped in its centre from a height of 1 cm. After 60 s, the spread of the water on the fabric surface is measured and reported in cm^2^. The fabric properties are summarized in Table 1 (single layers) and Table 2 (combinations).

**Figure 3:**
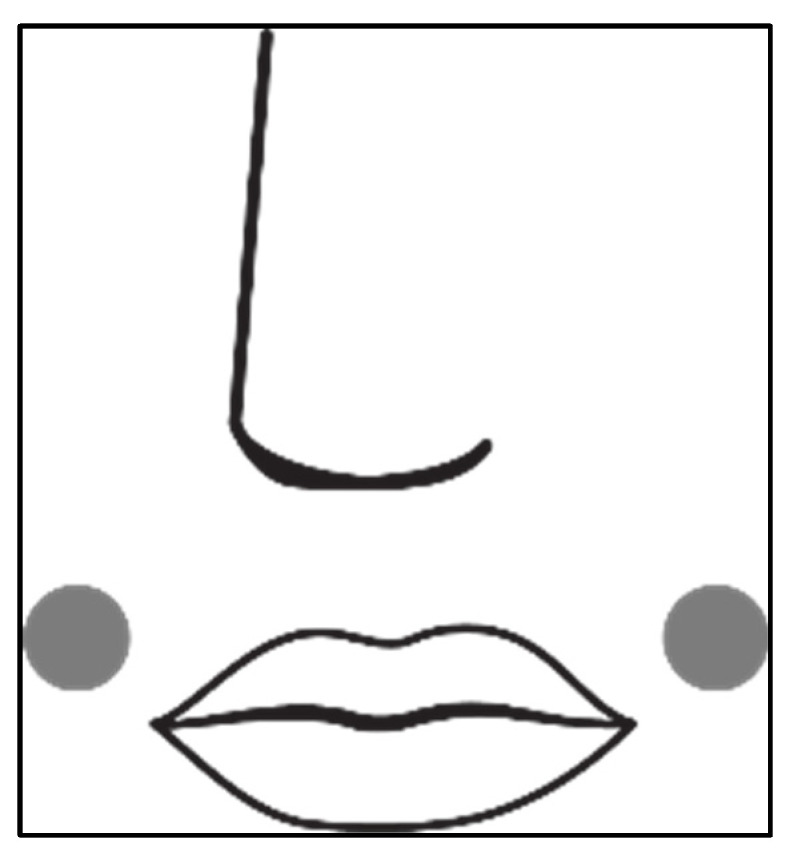
Illustration of placement of iButtons on the face.

Data were recorded digitally during the wear trials as explained. To minimize variances in the results, three precautions were implemented. Firstly, only one respondent was used to reduce physiological variances; secondly, the wear trials were carried out under controlled conditions (21 ± 0.5°C and 55 ± 5 % RH) to simulate office conditions; and, thirdly, every evaluation cycle was repeated five times. Before the start of each evaluation, the respondent was made comfortable and two iButtons were secured on the skin as shown in Figure 3. The positioning was selected to make sure the conditions in the micro-climate were recorded without being affected by warm or moist air expelled during breathing. The mask was then fitted securely on the face. The respondent was instructed to continue with sedentary work for at least 30 min while breathing normally through both the mouth and nose. The mask was not to be touched or adjusted unnecessarily. After every 10 min, a short (mild) exercise routine was followed. This comprised of moving or lifting the arms and legs in a controlled manner but making sure that the exercise did not lead to a change in breathing pattern. During the evaluation, the relative humidity and temperature inside the mask were measured continuously and recorded (logged) digitally as described.

Results were analysed using Tibco Statistica Software. The experimental design aimed to examine the effect of mask style versus that of mask components (fabrics) on the micro-climate between the skin and inner surface of the mask during wear. The main variables were mask style and fabric. The effect of fabric performance properties (such as moisture vapour permeability, wickability, thermal resistance) and fabric structural features (such as weight and thickness) were also investigated. The dependent variables were T_mc_ and H_mc_.

## 3. Results and Discussion

The temperature and humidity data were logged at a defined rate of one measurement every 60 s for 30 min, which was divided into three time segments, namely the first, second, and third 10 min of the trial (time segments are referred to, respectively, as TS1, TS2, and TS3). The results were analysed to identify the factors that can affect T_mc_ and H_mc_ during prolonged wear. Two-way analyses of variance (ANOVAs) were completed to determine the interactions between the factors of mask style and fabric structure and the T_mc_ and H_mc_. The initial statistical analyses showed significant differences between the measurements recorded within the different time segments, which indicated that the data should be analysed per time segment to prevent the overall means per factor from leading to false interpretations.

**Figure 4:**
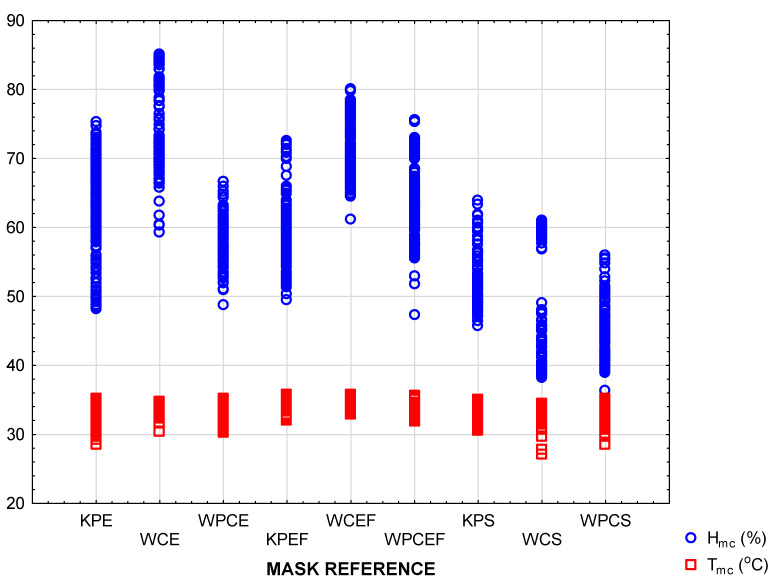
Scatterplot of multiple variates to provide an overview of all the data, depicted per mask design.

An overview of all the results is depicted in the scatterplots for T_mc_ and H_mc_ in Figure 4. The highest temperatures were measured inside the mask with an inserted filter (i.e., style EF masks). Concerning the humidity inside the mask, fabric type was the strongest determinant, with the highest values measured in the masks made of woven cotton.

### 3.1. Mask Style

**Figure 5:**
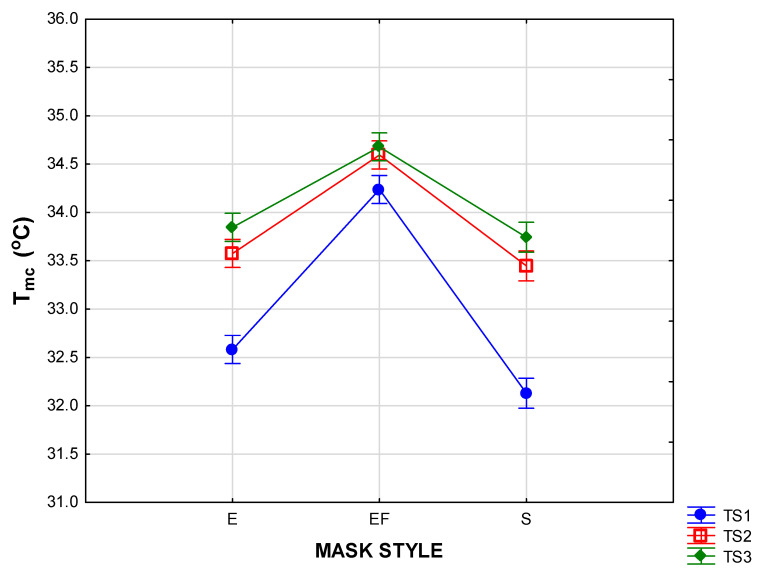
Two-way ANOVA of T_mc_ per mask style according to time segment.

**Figure 6:**
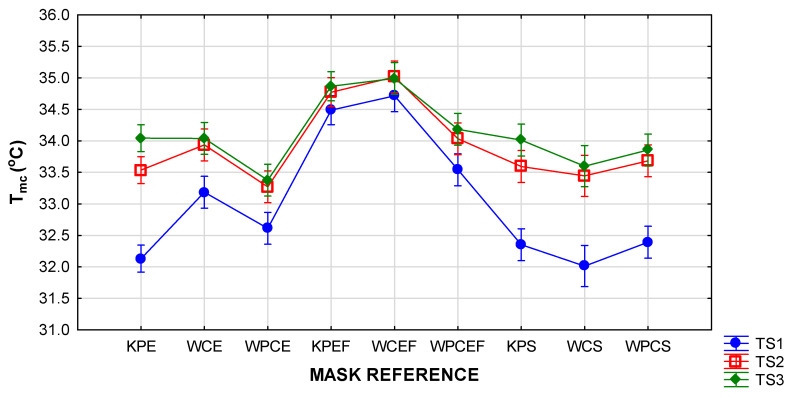
Two-way ANOVA of T_mc_ per time segment according to mask design (referenced as explained in Table 1).

**Figure 7:**
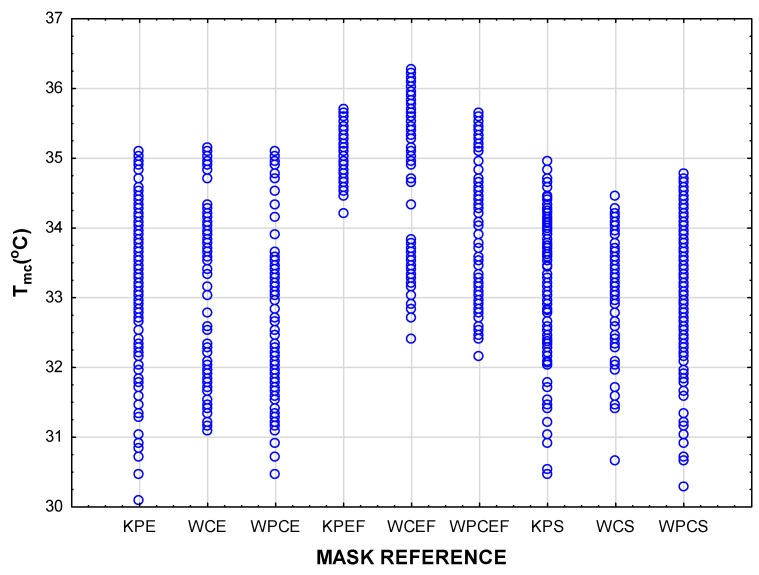
Scatterplot depicting individual values for the T_mc_ in all the mask designs.

**Figure 8:**
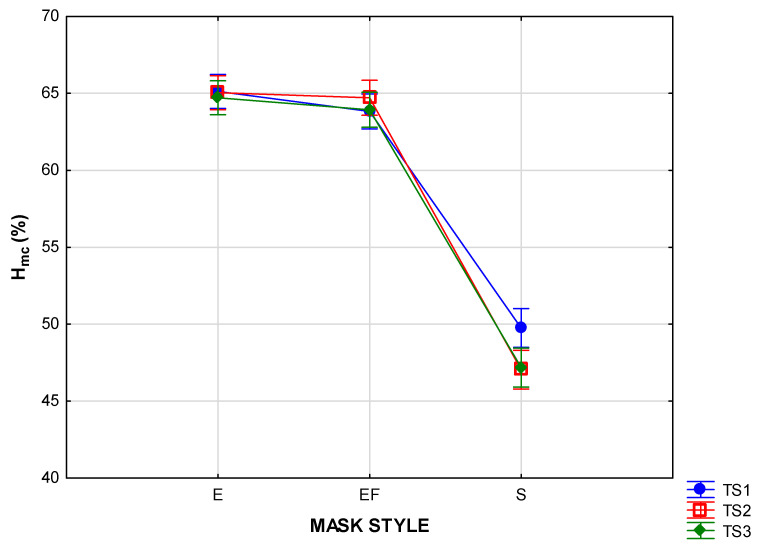
Two-way ANOVA of Hmc per mask style according to time segment.

The effects of the factors of mask style and time segment on T_mc_ and H_mc_ are depicted in the two-way ANOVAs in Figure 5, Figure 6, Figure 7 and Figure 8 (vertical bars denote 95 % confidence levels).

The interaction graph in Figure 5 confirms that the wearing of the pleated masks with the inserted filter (style EF) led to a significantly higher T_mc_ in the beginning and throughout the wear trials. The results were further interpreted in the interaction graph in Figure 6 to investigate the effect of mask design (combinations of mask style and fabric type) and time segments. The T_mc_ measurements in all the masks in this style exceeded those of the other styles at a highly significant level. This was expected as the physical properties of the inserted filter increased the thickness of the masks in this style to more than three times that of the others, and the laboratory measurements for thermal resistance (R_t_) on the three layers were more than three times higher than those of the fabrics in the other mask styles (Table 1). The remaining two mask styles (S and E) performed similarly in the last two time segments, but the temperature inside the shaped masks (style S) took significantly longer to stabilise, implying that a false perception could be formed regarding the comfort of the mask if the evaluation period is short.

Figure 6 indicates distinct differences in T_mc_ depending on when the measurements were taken. In most cases, the T_mc_ measurements in TS1 were much lower than those measured in TS2 and TS3, indicating a gradual increase during the time of wear. In both the pleated and shaped masks (styles E and S), the temperatures increased significantly in TS2 and TS3. The lowest initial T_mc_ measurements were recorded in the masks in style S (KPS, WCS, and WPCS) as well as one of the style E masks (KPE), but this was followed by a significant rise in T_mc_ after 10 min. In most cases, the rise between TS2 and TS3 was not significant. In all cases, the highest T_mc_ were recorded in the last 10 min of the wear trial. It should be noted that, in the style EF masks, the T_mc_ was high from the beginning and the increase in T_mc_ between the time segments was not significant. It can be noted from the scatterplot in Figure 7 that individual temperature measurements inside some of the style EF masks (predominantly in the woven cotton masks with reference WCEF) reached values exceeding 36 °C.

The differences in the thermal behaviour among the three mask styles are explained as follows: initially, the ambient temperature in the laboratory (21 °C) will determine the temperature inside the mask (T_mc_). However, during wear, the conduction of heat from the skin (temperature ± 36 °C) will lead to an increase in the temperature of the immediate air layer surrounding the face, which will then cause an increase in the temperature of the air in the micro-climate inside the mask. The increased temperature gradient will initiate the transfer of heat from the micro-climate to the environment through the fabric layers in the mask. The rate of transfer depends on the combined effect of the total amount of air involved and the thermal resistance of the fabrics in the masks. The combined effect of the air between the skin and the mask, as well as the air incorporated in the fabric structure and between the fabric layers, will contribute to the transfer of heat from the skin to the environment [9,14].

Due to the design of the shaped style S masks, the larger micro-climate in the mask causes a slower increase in T_mc_ than in the other styles. The “buffer-effect” caused by the prominent air layer against the skin slows down the conduction of heat from the skin surface. After 10 min, the buffer effect in the style S mask was overcome and the mask styles S and E performed similarly. The amount of air incorporated in the highly porous filter layer in mask style EF (without the effect of a “buffer air layer”) caused the development of a significantly higher temperature inside the micro-climate. This accords with the findings in the research on medical masks [21].

**Figure 9:**
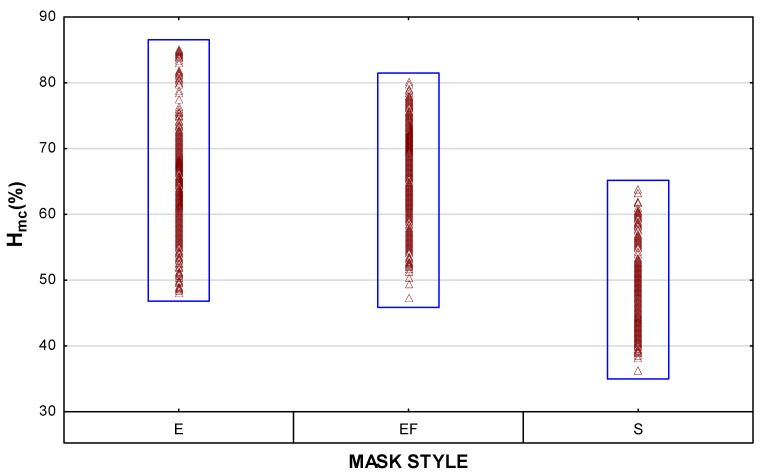
Variability plot of H_mc_ (raw data) per mask style.

**Figure 10:**
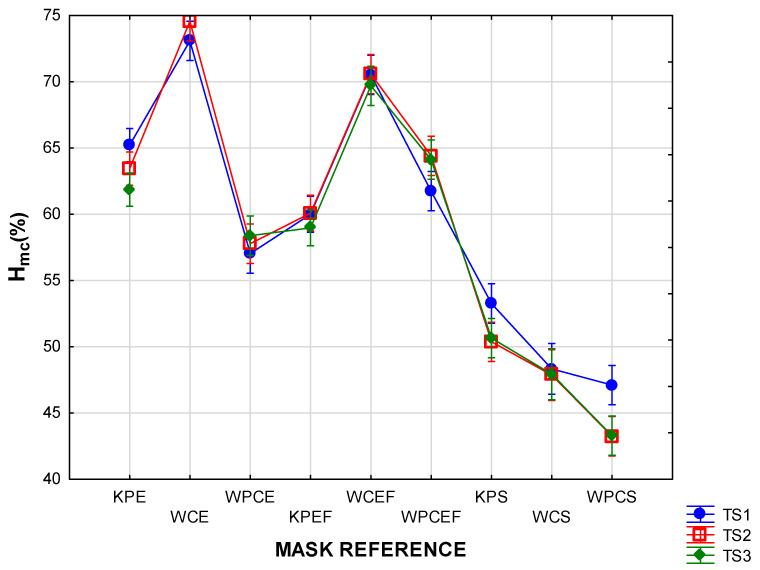
Two-way ANOVA of H_mc_ per time segment according to mask design (referenced as explained in Table 1).

The H_mc_ values measured inside the shaped masks (style S) were significantly lower than in the other two styles (*p* < 0.00). This is demonstrated in Figure 8, which also shows that the effect of the time segment was not significant in most of the masks in this style. It does appear, however, that, as with the T_mc_ results, the larger micro-climate in the shaped masks caused the H_mc_ levels to take longer to stabilise. Although the means for the style E and EF masks were very close, the variability plot (Figure 9) shows that some of the individual measurements in the style E masks were very high (> 80 %). This effect was further interpreted in Figure 10, where it is clear that fabric type plays a strong role in determining the properties of the style E masks.

### 3.2. Fabric Type

**Figure 11:**
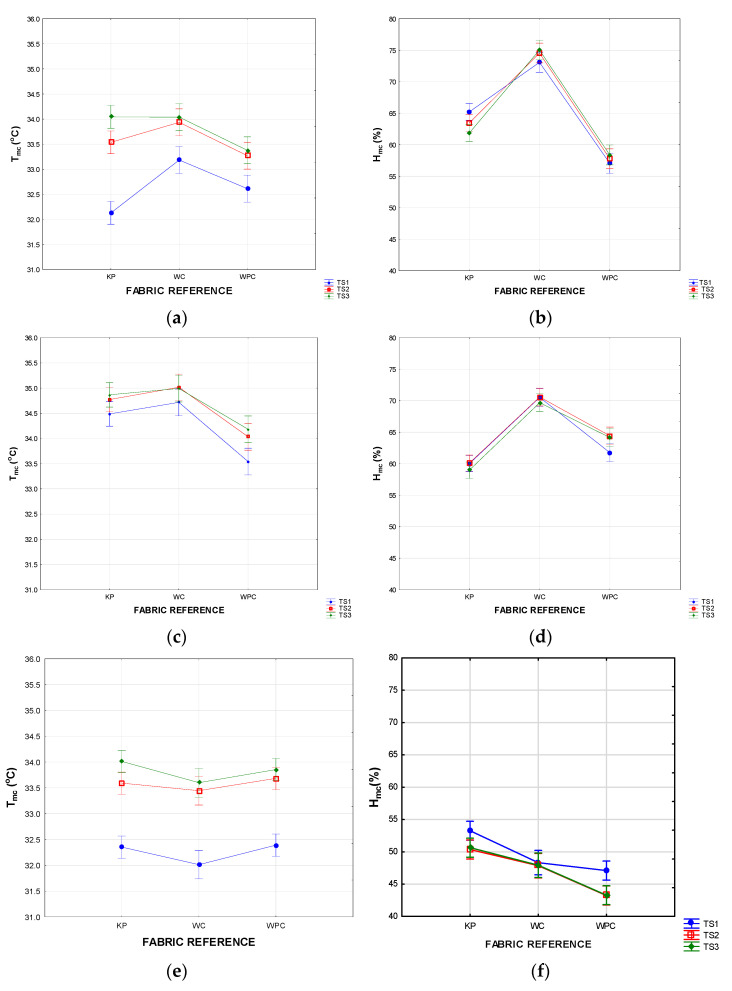
Two-way ANOVAs for T_mc_ and H_mc_ for all the mask styles interpreted per fabric type and time segment. (**a**) Pleated mask (style E); (**b**) pleated mask (style E); (**c**) mask with filter (style EF); (**d**) mask with filter (style EF); (**e**) shaped mask (style S); (**f**) shaped mask (style S).

The mask style had a significant effect on the performance of the fabrics tested. In the two-way ANOVAs in Figure 11a–f, the effect of fabric type (the textile fabrics used in the inner and outer layers) on T_mc_ and H_mc_ were further investigated. The results are interpreted separately for each mask style per fabric type and time segment. It should be noted that the fabrics do not only differ in structure and composition but also in thickness and weight and other performance properties (listed in Table 2).

When a mask fits snugly around the face, a thinner layer of air is expected in the micro-climate (compared to, for example, shaped masks), and the transfer of heat and moisture to the environment depend on the thermal and moisture management properties of the mask itself, which, in turn, depend on the collective performance properties of all the fabric layers in the mask. In the pleated masks (style E), the two fabric layers lie closely together, and the close fit of the mask minimizes the space between the skin and the mask. It is postulated that, in this type of mask, the T_mc_ and H_mc_ will be directly dependent on the specific thermal resistance (R_ct_) and moisture management properties of the fabrics in the mask. The effect of fabric type on T_mc_ and H_mc_ in the pleated masks (style E) is compared in the graphs in Figure 11a,b.

The weft-knit polyester fabric (type KP) is an eyelet knit design that incorporates a fair amount of still air and gives a soft drape to the fabric, allowing a snug fit around the face. Polyester fibres are known to exhibit a higher thermal resistance than cotton. This is confirmed in the R_ct_ measurements summarized in Table 2. The smooth filament fibres have an initial cool hand, and the good wicking propensity (as a result of a hydrophilic finish) will remove any liquid from the skin. This, together with the high air permeability that can cause heat loss through convection, could explain the initial low T_mc_ within the first 10 min. The wicking process will, however, allow moisture on the skin to move into the mask, which can decrease the effect of convection with time by filling up fabric interstices. The T_mc_ rises as heat is conducted from the skin to the thin layer of air in the micro-climate, and the low conductivity of the fabric layers resists further heat transfer to the outside environment. The knitted structure of the weft-knit polyester allows moisture vapour to escape to the environment as the humidity levels inside the mask start to increase. The concentration gradient between the micro-climate and the environment enhances the increase in the moisture vapour transfer rate, preventing the build-up of excess moisture vapour and causing the H_mc_ to decrease significantly from TS1 to TS3.

The woven cotton fabric (WC) is slightly thinner than the knitted polyester, and one would expect the fibres in the medium count woven structure to be incorporating less still air. The thermal conductivity of cotton is also higher than that of polyester [24]. This was confirmed in the Permetest results, where, as expected, the laboratory measurements for the thermal resistance (R_ct_) of two layers of WC were lower than those of KP and WPC (Table 2). The effect was, however, not noticeable in the masks. The thicker, woven design of the woven cotton made the fabric slightly stiffer than the knitted polyester and woven polycotton, trapping more air between the two fabric layers of the mask. The result is that, although better dissipation of heat was expected through the woven cotton, the T_mc_ in the pleated woven cotton masks ended up being almost the same as for those made of knitted polyester (Figure 11a).

The highest mean H_mc_ was measured in the pleated masks made of woven cotton (WC). The moisture vapour transfer through the woven cotton is less efficient than through the knitted polyester or woven polycotton, not only because of the thickness of the two layers but also because of the specific moisture management properties of the cotton fibres. Cotton has a moisture regain of 8% [24], and the fibres will absorb moisture from the skin, which will increase the moisture content in the first fabric layer and inhibit the transfer of moisture vapour, increasing the H_mc_. The results are also in line with the Permetest results in Table 1, where the water vapour permeability (WVP) for the Style E masks for WC, KP, and WPC are, respectively, 46.7%, 60.2%, and 52.9%. An increase in H_mc_ is noticeable from TS1 to TS3 for the WC masks in Figure 11b, confirming this postulation.

The T_mc_, as well as H_mc_, measured in the woven polycotton (WPC) masks is the lowest of the three fabric types. The effect was highly significant regarding H_mc_. The fabric has a high count, which allows less air to be incorporated into the fabric structure, explaining the R_ct_ of the WPC being the lowest of the three fabrics (8.5 m^2^·K.W^−^^1^). The low thickness of the fabric (0.2 mm) leads to a soft drape that allows a closer packing of the two layers in the mask and a tighter fit against the face. This incorporates less air (which could potentially increase the thermal resistance), and the soft drape of the mask allows heat and moisture vapour to escape from the micro-climate. The H_mc_ was significantly lower in the pleated masks containing WPC, which was a surprise considering that the water vapour permeability measurements of the knitted polyester were higher in both the single layer and double layer tests.

The T_mc_ in the pleated masks with the incorporated filters (style EF) was significantly higher than those in the pleated masks without filters (Style E) (Figure 5). The two styles are similar in design, but the incorporated filter layer in style EF adds to the thickness and stiffness of the mask. The graphs show clearly that the inclusion of the thicker nonwoven filter layer, which, by design, incorporates a substantial amount of still air, has a highly significant effect on the mask’s ability to dissipate heat from the micro-climate to the surrounding atmosphere (and, resultantly, on the T_mc_ in the case of all the fabric types), negating the effect of the individual fabric properties. The H_mc_ in all the style EF masks was, however, significantly lower than in the thinner and expectantly more permeable style E masks for fabrics KP and WC (Figure 8). This could be attributed to the increased stiffness of the EF masks, which caused them to fit less snuggly, allowing water vapour to escape through the open sides. This was not the case with the WPC fabric, which is softer and allowed a better fit around the face contours.

Concerning the shaped (style S) masks, the design, as well as the stiffer nonwoven fabric used in between the two layers of fabric, cause the front part of the mask to stand away from the face, resulting in a substantially larger micro-climate between the skin and the mask. The incorporation of the thicker layer of air provides increased resistance to the transfer of heat through the micro-climate to the fabric layers in the mask, confirming the results illustrated in Figure 8. The graph in Figure 11e confirms that the effect of fabric type in the style S masks is not significant in determining the regulation of the temperature inside the mask (*p* > 0.05).

The H_mc_ measured inside the style S masks (Figure 9) is visibly lower than those of style E and style EF (*p* < 0.00). As with T_mc_, this is caused by the larger micro-climate inside the mask. The H_mc_ will increase due to a build-up of water vapour from breathing and perspiration, but, as soon as the gradient between the concentration of moisture in the micro-climate and the ambient environment becomes bigger, moisture vapour transfer will start. The effect is further illustrated in the graph in Figure 11f, where a low H_mc_ is noted in the first 10 min, with a significant decrease towards the last two time segments in t of the fabric types. In the masks made of fabrics KP and WPC, this was confirmed by the increase in moisture vapour transfer after the first 10 min, reaching equilibrium in segments 2 and 3. This was not the case with the WC masks, where the ability of the cotton in the first layer to absorb moisture might have inhibited the process.

## 4. Conclusions

This study aimed to determine the effect of mask design and fabric type on the conditions inside the micro-climate between the skin and the mask. It was found that the mask style had a significant influence on the factors that determine mask performance: in the shaped masks, the design and fit caused a large air layer to form in the space between the skin and the mask. The impact of the air layer outweighed the effect of the fabric type on the T_mc_ and H_mc_ inside the mask. In the pleated masks with the inserted filter, the increase in the total mask thickness, together with the high porosity of the nonwoven filter, outweighed the effect of the fabric composition on mask comfort. In the pleated mask, the two fabric layers fit snugly around the face. This fit minimizes the air layer between the face and the mask and enhances the role that the fabric performance properties play in the effective transfer of heat and moisture from inside these masks to the environment.

The results indicate that, concerning T_mc_, it is evident that the amount of air incorporated between the skin and the mask significantly affects the rate at which body heat is transferred to the environment. This is highly noticeable in the shaped masks where the thicker air layer between the skin and the mask had an initial buffering effect on the increase in T_mc_ inside the micro-climate. In these masks, the impact of the larger micro-climate was stronger than the effect of fabric type.

The significant effect of the fabrics in the masks on T_mc_ is illustrated when the masks made of medium-weight woven cotton, knitted polyester, and lightweight woven polycotton fabrics are compared. The fibre composition, as well as fabric structure, both contributed to the differences found between the three fabrics. The highest T_mc_ was reported for the pleated-style masks with the inserted filter. This is due to the increase in thickness and incorporated still air (caused by the high porosity of the inserted filter).

Regarding the H_mc_ inside the masks, the larger micro-climate in the shaped masks inhibited the build-up of H_mc_, and, as with T_mc_, this outweighed the effect of fabric type. In the pleated-style masks, both with and without filters, the effect of fabric type was significant. The humidity levels inside the masks made of woven cotton fabric were the highest because of the absorbed moisture that inhibits the transfer of moisture vapour to the environment.

Significant differences were found between the temperatures recorded in the masks within the three time segments. In the folded and shaped masks, the T_mc_ measured in the first 10 min was much lower than that measured in the rest of the trial. The T_mc_ inside the shaped mask initially increased significantly slower than in the folded mask, but, in the second and third time sectors, the differences were not significant. This variance within the time intervals highlights how important it is that comfort evaluations are conducted for a long enough time to prevent an initial false interpretation. A mask may be perceived as comfortable if tested for a few minutes but may not perform as well in the long-run. None of the fabric structures or structural properties could be singled out as having a more pronounced influence than the others.

## Figures and Tables

**Table 1:** Referencing and description of structural and performance properties of fabric layer combinations resembling those in the masks selected for this study.

Mask Ref. #	Fabric Type	Filter Ref.	Mask Style	Weight (All Layers) (g/m^2^)	Thickness (All Layers) (mm)	WVP (%)	Rct (m^2^·K.W^−1^)	FE (%)	AP (%)
KPE	KP	none	E	260	1	60.2	22.6	91.0	95.0
WCE	WC	none	E	350	0.8	46.7	9.2	68.0	34.0
WPCE	WPC	none	E	250	0.4	52.9	15.4	76.0	30.0
KPEF	KP	Nw1	EF	360	6	29.8	156.9	90.0	84.0
WCEF	WC	Nw1	EF	450	5.8	21.3	92.7	91.0	31.0
WPCEF	WPC	Nw1	EF	350	5.4	26.9	129.9	97.0	16.0
KPS	KP	Nw2	S	310	1.6	44.0	50.5	72	80
WCS	WC	Nw2	S	400	1.4	37.0	28.3	72	21
WPCS	WPC	Nw2	S	300	1.0	45.0	27.9	92	16

# “Mask reference” describes the design of the mask and comprises combinations of three mask styles and three fabric types. The masks are referenced using the fabric reference, followed by the mask style (e.g., WCE = WC (woven cotton) + E (folded style mask)). Other abbreviations used include K (knitted), W (woven), Nw (nonwoven), P (polyester), C (cotton), and PC (poly-cotton).

**Table 2:** Structural and performance properties of (single layer) fabrics and filters used in the masks described.

Fabric Ref.	Fabric Structural Properties (Single Layer)				Fabric Performance Properties (Single Layer)				
	Fabric Structure	Fibre Content	Weight (g/m^2^)	Thickness (mm)	WVP (%)	R_ct_ (m^2^·K.W^−1^)	Wicking (cm^2^)	FE (%)	AP (%)
WC	Plain weave	Cotton	175	0.4	66.4	9.5	21.7	61	58
WPC	Plain weave	Poly-cotton	125	0.2	70.9	8.5	10.1	85	30
KP	Weft-knit (eyelet)	Polyester	130	0.5	76.7	10.3	24.0	69	97
Nw1	Fibre web	Polyester	100	5.0	30.3	117.0	*-*	75	92
Nw2	Fibre web	Polyester	50	0.6	67.8	24.1	*-*	68	90

## Data Availability

Not applicable.
